# Electrical Power Generation Using Dynamic Piezoelectric Shear Deformation Under Friction

**DOI:** 10.1007/s10338-021-00291-3

**Published:** 2021-11-26

**Authors:** Peng Wang, Yu Xiao, Nan Wu

**Affiliations:** 1 Department of Mechanical Engineering, Northeastern University, Shenyang, 110819 China; 2 Department of Mechanical Engineering, University of Manitoba, Winnipeg, R3T 2N2 Canada

**Keywords:** Electrical power generation, Friction, Piezoelectric shear deformation, Sliding and stick, Bi-linear

## Abstract

A new electrical power generation device based on high-frequency dynamic piezoelectric shear deformation under friction is developed. During the operation of a moving plate compressed and sliding on the top of a piezoelectric patch with constant velocity, dynamic shear deformation of the elastic piezoelectric patch is excited by periodic friction force and status (sliding and stick) variation. The dynamic piezoelectric shear strain can then generate continuous electrical power for energy absorbing and harvesting applications. The design of the piezoelectric couple device is first provided, and its mechanism, dynamic response and electric power generation under friction are described by a detailed iteration model. By comparing with previous experimental results, the accuracy of the proposed model is proven. Through numerical studies, the influences of the equivalent mass of the system, the velocity of the sliding object, the static friction coefficient and its lower limit, as well as the friction force delay rate on the power generation are obtained and discussed. The numerical results show that with the proposed design, up to 50-Watt maximum electrical power could be generated by a piezoelectric patch with a dimension of $20\times 2\times 6$ cm under continuous friction with the moving plate at the velocity of 15 m/s. The possible bi-linear elastic stiffness variation of the system is also introduced, and the threshold of bi-linear elastic deformation, where the system stiffness changes, can be optimized for obtaining the highest power generation.

## Introduction

With the power demand for wireless electronic devices and the increasingly wide application of sensors in different structures, energy harvesting based on piezoelectric materials has become an active research field, which has attracted wide attention in recent years [1–3].

Since the piezoelectric shear constant is usually higher than that along the normal directions [4], many scholars aimed to realize energy harvesting by making good use of piezoelectric materials’ shear deformation [5–9]. In addition, there are also some research on the piezoelectric prediction model, the derivation and solution of the electromechanical governing equation, and the calculation and optimization of the output voltage of piezoelectric shear cantilever beams [10–14].

Friction is a commonly existing physical phenomenon in engineering applications [15, 16]. In recent years, the structural vibration characteristics under friction, including the transformation between dynamic and static frictions [17], system stability analysis [18, 19], and energy harvesting under friction [20, 21] have received attention from researchers. Studies have been conducted on vibration response [22–24] and energy harvesting from friction [25–30]. Li et al. solved the final wear shape of elastic indenter by the dimension reduction method. The results showed that the vibration amplitude and the initially indented or moved displacements influence the final mode shape [22]. Li et al. considered the cubic contact spring, which allowed the contact loss (separation) at the slider belt interface and studied the reconnection of the slider and belt after separation. These two characteristics make the friction-induced vibration model more realistic [23]. Xing et al. used friction-induced vibration to identify the friction state in the lubrication friction system [24]. As long as the friction-induced vibration response can be obtained, the energy harvesting under dynamic loads/deformation can be realized. Green et al. studied the inevitable nonlinear problems of an electromagnetic energy harvester utilizing friction [25]. Wang et al. proposed a nonlinear two-degree-of-freedom friction system with piezoelectric elements considering the stick-sliding motion, model coupling instability, mass and belt separation and reattachment simultaneously. The complex eigenvalue analysis and transient dynamic analysis of the nonlinear system were carried out [26]. Tadokoro et al. proposed an analytical model of a single-degree-of-freedom system with friction and piezoelectric elements to study the vibrational response under friction, and the model was verified by experiment [27]. Li et al. proposed a piezoelectric energy harvester based on spring mass oscillator. The results showed that the harvester can output high voltage at resonant frequency [28]. Chen et al. sandwiched the piezoelectric plate between two layers of elastic damping elements and proposed a method to realize energy harvesting through frictional vibration and vibration reduction at the same time [29]. Wang et al. proposed a single-degree-of-freedom piezoelectric friction system and studied the effect of stick-sliding vibration caused by friction on piezoelectric energy acquisition. On this basis, a two-degree-of-freedom piezoelectric friction system was also proposed, and the problem of piezoelectric energy harvesting via mode coupling vibration was studied [30]. The above research included different frictional vibration models, numerical analyses and experimental studies on piezoelectric energy harvesting under friction. However, according to the author’s knowledge, there is no research which describes the piezoelectric dynamic shear deformation under friction and helps people to understand its possible high operation frequency and application on electrical power generation.

In this paper, a new simple piezoelectric couple device under piezoelectric shear deformation with high stiffness is designed to convert low-frequency excitation to high-frequency (close to the acoustic range) structural response using friction as excitation. The design has the potential high power generation making good use of both high piezoelectric shear coefficient and friction-induced high-frequency vibration. A clear and simple theoretical model is proposed, and through numerical simulations, we can describe, explain, discuss and study the high-frequency dynamic response and high piezoelectric power generation of the proposed structure operated under high sliding velocity and large friction force. On this basis, bi-linearity of the elastic element stiffness [31–33] is also introduced to the friction model, and parameter analysis is conducted, leading to an optimal design of the proposed friction energy harvesting device.

## Method and Modeling

**Fig. 1:**
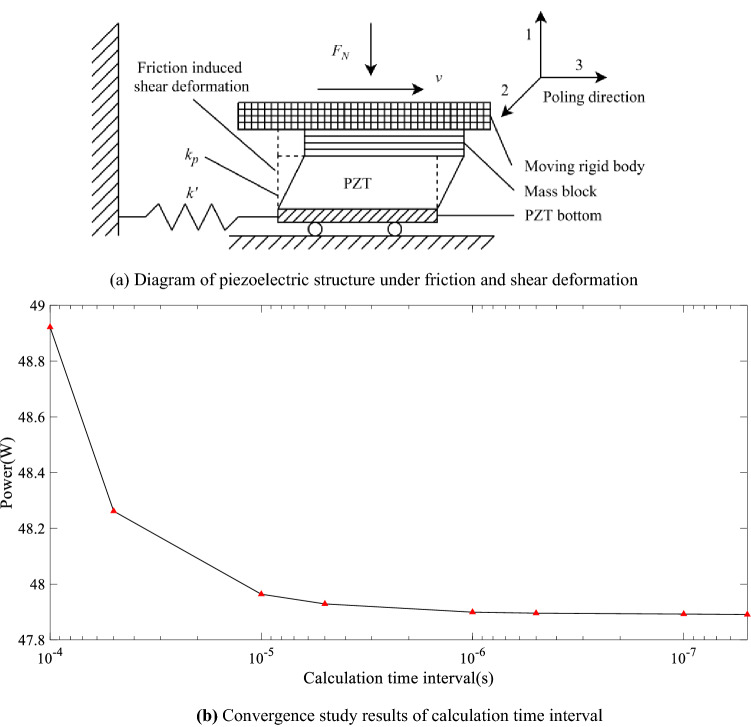
Piezoelectric structure and convergence study

A single-degree-of-freedom piezoelectric shear device studied in this paper is shown in Fig. 1a. On the top of the piezoelectric patch (lead zirconate titanate (PZT) is used in this work), a rigid mass block is attached. A rigid moving plate is contacting with the mass block and moving at a constant velocity horizontally. At its bottom, the piezoelectric material is held by an elastic clamp, which can be considered as a tension spring as shown in the figure. The whole PZT block can move along the horizontal direction at its bottom and be subjected to shear deformation at the same time. It should be noted that in reality, we need to consider different surface conditions of the

piezoelectric patch and mass block, such as rough or serrated surface, to enhance the interface bonding. The shape of the electrode will follow the piezoelectric surface profile, leading to larger electrode area and possible higher charging amount. In this case, the tension spring and the shear-deformable PZT can be considered as two springs connected in series with a mass block located at their free end, making a single-degree-of-freedom spring-mass system. *m* is the equivalent mass of the tension spring, PZT, and the mass block at the free end of the whole elastic elements. $$k'$$ is the stiffness constant of the tension spring. $$k_{p} $$ is the stiffness constant of PZT.

The equivalent stiffness of the system can be written as $$k=k_{p} \cdot k'/(k_{p} +k')$$ considering the spring and the PZT with shear deformation connected in series. $$F_{N} $$ is the normal pressure acting on the plate along the vertical direction. *v* is a constant moving velocity of the top moving plate, while the moving direction to the right is defined as positive in this work.

With the normal pressure and nonzero friction coefficient, when the plate is sliding on the top of the mass block, friction force will be generated between the moving plate and the mass block. If we assume that the mass block is initially moving with the sliding plate at velocity, *v*, from the equilibrium position of the single-degree-of-freedom system, the mass block moves towards the right of the system with the ‘stick’ friction state first until the spring force is higher than the largest static friction force. The high spring force will then lead to the ‘rebound’ of the mass block and ‘sliding’ friction status, and in this case, the friction force applied on the mass becomes dynamic sliding friction force. When the mass block is moving back during the ‘rebound’ process and its velocity catches up the velocity of the moving plate with the spring force smaller than the maximum static friction, the mass sticks with the plate again, and the static stick friction force equals to the current spring force before the next ‘rebound’ phenomenon. This whole process induces dynamic friction force applied on the mass block and its vibration as well as the reciprocating dynamic shear deformation on the PZT element to generate electrical energy. In the following section, the detailed model and iteration process describing the friction induced vibration of the single degree of freedom PZT shear system is provided.

### Friction Model and Vibration of Single-Degree-of-Freedom System

The vibration equation of piezoelectric shear device under friction force is,

1 $$\begin{aligned} m\ddot{{x}}(t)+c\dot{{x}}(t)+kx(t)=f(t) \end{aligned}$$

where *c* is the damping coefficient, $c=2m\omega \xi$, $\xi$ is the damping ratio, $\omega$ is the natural frequency, *f*(*t*) is the friction force applied to the mass block, and *x*(*t*) is the vibration response of block under friction force.

The premise of the iterative method is that at $$t=$$0 s, the dynamic initial conditions are known. The step length of each iteration is $$\Delta t=t_{n+1} -t_{n} $$, $$1\le n<\infty $$, and the subscript *n* is the number of iteration steps. The subscript $$n=$$1 represents the calculation in the first time period/iteration step in period $$t_{1} $$-$$t_{2} $$. The subscript $$n=$$2 represents the second time period/iteration step in period $$t_{2} $$-$$t_{3} $$, and so on. The subscript *n* represents the *n*-th time period/iteration step in period $$t_{n} $$-$$t_{n+1} $$.

The initial condition for time $$t_{1} $$ ($$t_{1} =$$0 s) is,

2 $$\begin{aligned} \left\{ {\begin{array}{l} x_{1} (t_{1} )\text{= }0 \\ \left. {\frac{\text{ d }x_{1} (t)}{\text{ d }t}} \right| _{t\text{= }t_{\text{1 }} } \text{= }v \\ \end{array}} \right. \end{aligned}$$

where $x_{1} (t_{1} )$ is the initial displacement of the block at the equilibrium position of the single-degree-of-freedom system.

Again, if we assume that the mass *m* in Fig. 1a, initially moves with the top plate at velocity *v* in the period $$t_{1} $$-$$t_{2} $$, the stick friction status with static friction force applied on the top of the mass should be considered. The displacement response of the mass block in $$t\text{= }t_{1} -t_{2} $$ is,

3 $$\begin{aligned} x_{1} (t)=v\cdot t~(t_{1} \le t\le t_{2} ) \end{aligned}$$

The stick friction force applied to the mass block is,

4 $$\begin{aligned} f_{1} (t)=x_{1} (t)\cdot k~(t_{1} \le t\le t_{2} ) \end{aligned}$$

which is along the opposite direction of the spring force but with the same absolute value. In the second iteration period    $$t_{2} $$-$$t_{3} $$, if the absolute value of the spring force is $$\left| {x_{1} (t_{2} )\cdot k} \right| \le F_{N} \cdot \mu _{S} $$, ($$\mu _{S} $$ is the static friction coefficient), the contact state between block and plate during the current time period is still stick, and the dynamic response of the mass block is,

5 $$\begin{aligned} x_{2} (t)=v\cdot (t-t{ }_{2})+x_{1} (t_{2} ) ~(t_{2} \le t\le t_{3} ) \end{aligned}$$

The friction force applied to the mass block is,

6 $$\begin{aligned} f_{2} (t)\text{= }k\cdot x_{2} (t) ~(t_{2} \le t\le t_{3} ) \end{aligned}$$

On the other hand, if $$\left| {k\cdot x_{1} (t_{2} )} \right| >F_{N} \cdot \mu _{S} $$, the contact state between block and plate during the current period is sliding. By assuming no significant velocity variation in a short iteration period, the sliding friction force applied to the mass block in the period $$t=t_{2} -t_{3} $$ is [17],

7 $$\begin{aligned} f_{2} (t)=\mathrm{sgn}(v-\dot{{x}}_{1} (t_{2} ))\cdot \mu _{d} (v-\dot{{x}}_{1} (t_{2} ))\cdot F_{N} ~(t_{2} \le t\le t_{3} ) \end{aligned}$$

where $$\mu _{d} (X)=\mu _{D} \text{+( }\mu _{S} -\mu _{D} \text{)e }^{\text{- }C\left| X \right| }$$ is the dynamic friction coefficient, and we have $$X=v-\dot{{x}}_{1} (t_{2} )$$ in the period $$t_{2}~-~t_{3} $$. $$\mu _{D} $$ is the lower limit of $$\mu _{S} $$, and *C* is the delay rate of relative sliding velocity control coefficient between the block and plate.

The vibration response during the current time period is

8 $$\begin{aligned} \begin{array}{l} x_{2} (t) ={x}_{{free}_{2}} (t)\text{+ }{x}_{{force}_{2}} (t)\text{=e }^{-\xi \omega t}[(A_{2} \cos \omega _{d} t+B_{2} \sin \omega _{d} t) \\ \qquad +\frac{1}{m\omega _{d} }\int _0^{t-t_{2} } {f_{2} } (\tau +t_{2} )\text{ e}^{-\xi \omega (t-t_{2} -\tau )}\cdot \sin (\omega _{d}(t-t_{2} -\tau ))\text{ d }\tau ] \\ \end{array}~(t_{2} \le t\le t_{3} ) \end{aligned}$$

where $$A_{2} $$ and $$B_{2} $$ are determined by the initial conditions, $$x_{1} (t)$$ and $$\dot{{x}}_{1} (t)$$, from the previous iteration step at $$t=t_{2} $$

9 $$\begin{aligned}&\text{ e}^{\text{- }\xi \omega t_{2} }(A_{2} \cos \omega _{d} t_{2} +B_{2} \sin \omega _{d} t_{2} )\text{= }x_{1} (t_{2} ) \end{aligned}$$

10 $$\begin{aligned}&\quad \text{ e}^{\text{- }\xi \omega t_{2} }\left[ {\cos \omega _{d} t_{2} (B_{2} \omega _{d} \text{- }\xi \omega A_{2} )-\sin \omega _{d} t_{2} (B_{2} \xi \omega +A_{2} \omega _{d} )} \right] =\dot{{x}}_{1} (t_{2} ) \end{aligned}$$

leading to

11 $$\begin{aligned}&B_{2} \text{= }\frac{\text{ e}^{\xi \omega t_{2} }(\cos \omega _{d} t_{2} (\xi \omega x_{1} (t_{2} )+\dot{{x}}_{1} (t_{2} ))+\omega _{d} x_{1} (t_{2} )\sin \omega _{d} t_{2} )}{\omega _{d} } \end{aligned}$$

12 $$\begin{aligned}&\quad A_{2} \text{= }\frac{1}{\cos \omega _{d} t_{2} }\text{( }x_{1} (t_{2} )\text{ e}^{\xi \omega t_{2} }-B_{2} \sin \omega _{d} t_{2} ) \end{aligned}$$

Following the similar idea, when the time is in the period $$t_{n} $$-$$t_{n+1} $$, the general friction force and dynamics response are calculated as follows.

For the sliding contact state of the block and plate in the previous period, if $\dot{{x}}_{n-1} (t_{n} )\text{= }v$ and $\left| {k\cdot x_{n-1} (t_{n} )} \right| \le F_{N} \cdot \mu _{s}$, the contact state between block and plate during the current time period becomes stick. The total dynamic response is,

13 $$\begin{aligned} x_{n} (t)=v(t-t_{n} )+x_{n-1} (t_{n} )~(t_{n} \le t\le t_{n+1} ) \end{aligned}$$

The friction force applied to the mass block is,

14 $$\begin{aligned} f_{n} (t)=k\cdot x_{n} (t)~(t_{n} \le t\le t_{n+1} ) \end{aligned}$$

If $\dot{{x}}_{n-1} (t_{n} )\ne v$ or $\left| {k\cdot x_{n-1} (t_{n} )} \right| >F_{N} \cdot \mu _{s}$, the contact state between block and plate during the current time period is still sliding. The friction force applied to the mass block is,

15 $$\begin{aligned} f_{n} (t)\text{= }\mathrm{sgn}(v-\dot{{x}}_{n-1} (t_{n} ))\cdot F_{N} \cdot \mu _{d} (v-\dot{{x}}_{n-1} (t_{n} ))~(~t_{n} \le t\le t_{n+1}~) \end{aligned}$$

The total vibration response during the time period $$t_{n} $$-$$t_{n+1} $$ is,

16 $$\begin{aligned} \begin{array}{l} x_{n} (t)\text{= }x_{free_{n} } (t)\text{+ }{x}_{{force}_{n}} (t)\text{=e }^{-\xi \omega t}[(A_{n} \cos \omega _{d} t+B_{n} \sin \omega _{d} t) \\ +\frac{1}{m\omega _{d} }\int _0^{t-t_{n} } {f_{n} } (\tau +t_{n} )\text{ e}^{-\xi \omega (t-t_{n} -\tau )}\cdot \sin (\omega _{d} (t-t_{n} -\tau ))\text{ d }\tau ] \\ \end{array} (t_{n} \le t\le t_{n+1} ) \end{aligned}$$

where $$A_{n} $$ and $$B_{n} $$ are determined by $$x_{n-1} (t)$$ and $$\dot{{x}}_{n-1} (t)$$ at $$t=t_{n} $$ from the previous iteration step, leading to

17 $$\begin{aligned}&A_{n} \text{= }\frac{1}{\cos \omega _{d} t_{n} }\text{( }x_{n-1} (t_{n} )\text{ e}^{\xi \omega t_{n} }-B_{n} \sin \omega _{d} t_{n} ) \end{aligned}$$

18 $$\begin{aligned}&\quad B_{n} \text{= }\frac{\text{ e}^{\xi \omega t_{n} }(\cos \omega _{d} t_{n} (\xi \omega x_{n-1} (t_{n} )+\dot{{x}}_{n-1} (t_{n} ))+\omega _{d} x_{n-1} (t_{n} )\sin \omega _{d} t_{n} )}{\omega _{d} } \end{aligned}$$

On the other hand, for the stick contact state of the block and plate in the previous period, if $\left| {k\cdot x_{n-1} (t_{n} )} \right| \le \mu _{s} F_{N}$, the contact state between mass block and plate during the current time period is still stick. The dynamic response and friction force between block and plate are solved by Eqs. (13) and (14), respectively.

If $\left| {k\cdot x_{n-1} (t_{n} )} \right| >\mu _{s} F_{N}$, the contact state between mass block and plate during the current time period becomes sliding. The friction force on the mass block is,

19 $$\begin{aligned} f_{n} (t)\text{= }F_{N} \cdot \mu _{d} (v-\dot{{x}}_{n-1} (t_{n} ))~(t_{n} \le t\le t_{n+1} ) \end{aligned}$$

The total vibration displacement during an arbitrary time period $$t_{n} $$-$$t_{n+1} $$ is,

20 $$\begin{aligned} \begin{array}{l} x_{n} (t)\text{= }x_{free_{n} } (t)\text{+ }x_{{force}_{n}} (t)\text{=e }^{-\xi \omega t}[(A_{n} \cos \omega _{d} t+B_{n} \sin \omega _{d} t) \\ \qquad +\frac{1}{m\omega _{d} }\int _0^{t-t_{n} } {f_{n} } (\tau +t_{n} )\text{ e}^{-\xi \omega (t-t_{n} -\tau )}\cdot \sin (\omega _{d} (t-t_{n} -\tau ))\text{ d }\tau ] \\ \end{array}~(~t_{n} \le t\le t_{n+1} ) \end{aligned}$$

with the same $$A_{n} $$ and $$B_{n} $$ defined in Eqs. (17) and (18).

The elastic bilinear system means that when the displacement of mass is greater than $$x'$$, the equivalent stiffness *k* becomes $$k_{E} $$. When $$x(t)>x'$$, the vibration equation of piezoelectric shear device under friction force is,

21 $$\begin{aligned} m\ddot{{x}}(t)+c\dot{{x}}(t)+kx'+k_{E} (x(t)-x')=f(t) \end{aligned}$$

Bilinear and single systems belong to different systems, and their solving processes of vibration response under friction force are the same. It is noted that all of the derived displacement responses above are the total deformation of the elastic spring elements (PZT and its clamp connected in series) from the equilibrium position of the system. Considering the stiffness distribution of $$k'$$ and $$k_{p} $$ in the series-connected spring, the PZT deformation during an arbitrary time period $$t_{n} $$-$$t_{n+1} $$ can be derived as

22 $$\begin{aligned} \forall x_{n} (t)=x_{n} (t)\cdot k/k_{p}~(t_{n} \le t\le t_{n+1} ) \end{aligned}$$

Once the dynamic deformation on the PZT patch due to friction can be derived, the electrical power generation can be solved based on the linear piezoelectric constitutive equation.

### Power Generation by Friction

Equation (22) gives the exact dynamic deformation happening on the PZT patches shown in Fig. 1a. It should be noted that the deformation given in Eq. (22) is derived and measured from the equilibrium position of the system, which could be different from the balance position of the PZT vibration with certain shift. In this case, considering the self-discharging of piezoelectric materials under static deformation, the charge, voltage, and power generation from the piezoelectric patch should be calculated based on its actual vibration deformation, $\Delta x(t)=\forall x_{n} (t)-Dx$, where *Dx* is the mean value of $\forall x_{n} (t)$ during its steady state.

In this work, we consider a piezoelectric patch with the poling direction along direction 3 as shown in Fig. 1a. The PZT is under shear deformation subjected to the friction along direction 3. The electrodes are located perpendicular to the 1-axis on the top and bottom surfaces of the PZT patch. The electric charging density on the PZT electrodes under shear deformation in the 31-plane is given below,

23 $$\begin{aligned} D_{1} =d_{15} \tau _{31} +\varepsilon _{11} E_{1} \end{aligned}$$

where $$D_{1} $$ is the surface charge density displacement along direction 1; $$d_{15} $$ is the shear piezoelectric charge coefficient; $$\tau _{31} $$ is the shear stress in the 31-plane; $$\varepsilon _{11} $$ is the permittivity for dielectric displacement and electric field in direction 1; and $$E_{1} $$ is the external applied electric field in direction 1. Since it is assumed that there is zero applied electric field through direction 1, the surface charge density from shear stress in the 31-plane is

24 $$\begin{aligned} D_{1} =d_{15} \tau _{31} \end{aligned}$$

The total surface charges $$Q_{g} $$ due to the PZT shear deformation ($$\Delta x)$$ at time*t* is solved by

25 $$\begin{aligned} Q_{g} (t)=D_{1} A=D_{1} lw=d_{15} \tau _{31} lw=d_{15} \frac{k_{p} \Delta x(t)}{lw}lw=d_{15} k_{p} \Delta x(t) \end{aligned}$$

Considering an equivalent plate capacitor with the same capacitance of the piezoelectric patch under the same charge on its electrodes, the generated voltage should be

26 $$\begin{aligned} V_{g} (t)=\frac{D_{1} A}{C_{v} }=\frac{d_{15} k_{p} \Delta x(t)}{C_{v} } \end{aligned}$$

where *l*, *w*, and *h* are the length, width, and thickness of PZT patch, respectively; and *A* is the surface area of the piezoelectric patch,

27 $$\begin{aligned} A=l*w \end{aligned}$$

$$C_{v} $$ is the electric capacity of the piezoelectric patch capacitor in Farad, which can be solved by

28 $$\begin{aligned} C_{v} =\frac{K^\mathrm{T}_{33} \varepsilon _{0} lw}{h} \end{aligned}$$

where $K^\mathrm{T}_{33}$ is the relative dielectric constant of the piezoelectric material; and $\varepsilon _{0} =8.85\times 10^{\text{- }12}$ farad per meter (F/m) is the permittivity of free space constant.

The stiffness constant of PZT ($$k_{p} )$$ can be represented by

29 $$\begin{aligned}&k_{p} =G_{13} \frac{A}{h} \end{aligned}$$

30 $$\begin{aligned}&\quad G_{13} =\frac{\gamma _{1} }{2(1+\nu _{13} )} \end{aligned}$$

where $$G_{13} $$ is the shear modulus of the piezoelectric material, $$\gamma _{1} $$ is the Young’s modulus in direction 1, and $$\nu _{13} $$ is the Poisson’s ratio.

Then, the electrical power input to charge the equivalent plate capacitor and equivalent maximum output by the piezoelectric effect, $P_{\text{ e }} (t)$, can be solved,

31 $$\begin{aligned} P_{\text{ e }} (t)=\frac{\text{ d }Q_{g} (t)}{\text{ d }t}V_{g} (t) \end{aligned}$$

The RMS (root mean square) value of the maximum generated power time 0 to *T* is given as:

32 $$\begin{aligned} P_{\text{ e }}^{rms} =\sqrt{\frac{1}{T}\int {\left[ {P_{\text{ e }} (t)} \right] }^{2}\text{ d }t} \end{aligned}$$

With the discrete displacement and power generation data, to estimate the RMS of the generated power, *T* can be separated into *j* time steps with a short time interval $\Delta t$. As a result, the expression in Eq. (32) can be rewritten in a discrete form below:

33 $$\begin{aligned} P_{\text{ e }}^{rms} =\sqrt{\frac{\Delta t}{2(T-\Delta t)}\sum \limits _{i=2}^j {\left( {\left[ {P_{\text{ e }} (t_{i} )} \right] ^{2}+\left[ {P_{\text{ e }} (t_{i-1} )} \right] ^{2}} \right) } } \end{aligned}$$

## Numerical Studies, Results and Discussion

In this section, the proposed numerical model is first validated by comparing with previous experimental results using the same system parameters. The time interval in the iterative method is first determined by a convergence study at the highest operation frequency of 123 Hz. The operation frequency is the fundamental frequency (or stick-slip frequency) of the friction-induced vibration, which is close to the natural frequency of the proposed system. The results are shown in Fig. 1b (the x-axis is represented by a logarithmic coordinate with a base of 10), leading to the iteration time interval of 0.000001 s to ensure the convergence.

### Model Validation

**Fig. 2:**
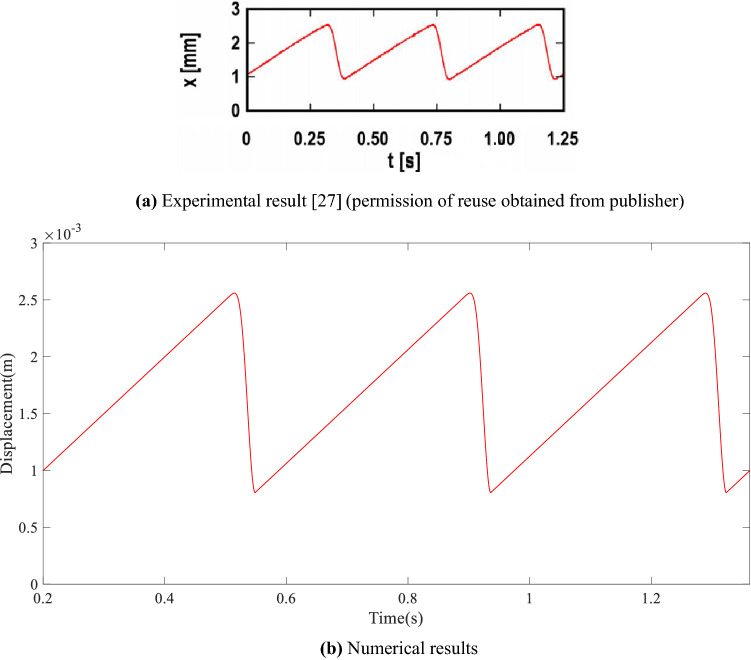
Comparison between experimental data and calculation results ($v=$0.005 m/s, $k=$890 N/m, $m=$0.08 kg, $F_{N} =$15 N, $\mu _{S} =$0.15, $\mu _{D} =$0.0885, $\xi =$0.0046, and $C=$22)

In order to verify the friction model in this paper, numerical results are compared with the experimental data and numerical result in a previous study [27] on a similar spring mass system vibration under friction. Among them, Fig. 2a is the dynamic displacement of the mass of a single-degree-of-freedom vibration system under friction measured from physical tests, and Fig. 2b is the calculation of the dynamic displacement in this paper considering the same parameters.

The start time point at $t=$0 s in this research corresponds to the initial location of the vibration system at its static equilibrium position, while the time point of $t=$0 s in the experimental results of reference [27] is manually selected/defined, close to the start point of a full vibration period under friction. To avoid misunderstanding, the vibration response starting from a full vibration period at 0.2 s obtained from this work is shown in Fig. 2b. It can be seen from the comparison with the data in reference [27] that the calculation results by the proposed model (of which the amplitude and period are 0.87745 mm and 0.3876 s, respectively) are closer to the experimental results (of which the amplitude and period are 0.75 mm and 0.417 s, respectively) than the numerical results in reference [27] (of which the amplitude and period are 0.8 mm and 0.37 s, respectively), which proves the correctness of the friction model. In our model, a more realistic sliding friction force model is developed considering the possible force drop delay when the friction state changes from stick to sliding.

### Parameter Studies

**Fig. 3:**
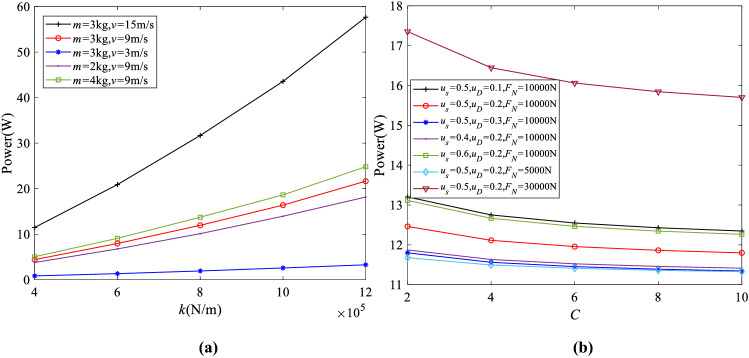
**a** Influences of *k*, *m*, *v* on power generation ($$F_{N} =$$10000 N, $$\mu _{S} =$$0.5, $$\mu _{D} =$$0.2, $$\xi =$$0.001, and $$C=$$6); **b** Influences of $$\mu _{S} $$, $$\mu _{D} $$, *C* and $$F_{N} $$ on power generation ($$v=$$9 m/s, $$k=0.8\times 10^{6}$$N/m, $$m=$$3 kg, and $$\xi =$$0.001)

The mechanical and electrical material properties and dimensions of the piezoelectric patch (APC 850) used in the numerical calculation are set to be: $K^\mathrm{T}_{33} =1900$, $d_{15} =5.9\times 10^{-10}$ C/N, $\gamma _{1} =6.3\times 10^{10}$ N/m$^{2}$, $\nu _{13} =0.36$, $l=0.2$m, $w=0.02$ m and $h=0.06$m. In order to study the influences of different parameters on the power generation of the system, Fig. 3a shows the variation trend of the power generation of the system with different *k*, *m* and *v*. The changing ranges of *k*, *m* and *v* are $0.4\times 10^{6}$-$1.2\times 10^{6}$ N/m, 2-4 kg and 3-15 m/s, respectively. Other parameters remain unchanged as described in the caption of Fig. 3a, and this set of structural parameters is selected for the energy harvesting with high structural stiffness and high operation frequency. The damping ratio of 0.001 is selected considering small damping effect of the proposed system. It is noted that in the whole process of motion, the electrical power generated by piezoelectricity (11.96 W) is actually much less than the energy absorbed by the assumed damping effect (173.6 W), proving the feasibility of the power generation reported in this work with the intermediate value of each parameter. From Fig. 3a, it can be seen that the power generation of the system increases with the increase of equivalent spring stiffness *k*, when *k* varies from $0.4\times 10^{6}$ to $1.2\times 10^{6}$ N/m. Within the ranges of the parameters studied, the power generation can be increased up to 5.16 times with parameters, $m=$3 kg and $v=$15 m/s. With other parameters unchanged, the increments of *m* and *v* lead to higher system power generation. The power can be increased by up to 17 times when *v* increases by 5 times with constant parameters, $m=$3 kg and $k=1.2\times 10^{6}$ N/m. Therefore, it can be concluded that the velocity of the sliding object has the greatest impact on the power generation of the system. On the other hand, it is not difficult to see that among the three parameters studied, *m* has the least influence on the power generation of the system. In our work, one of the reasons for the large difference in power generation is that the stiffness, the volume of piezoelectric material and the working frequency of the proposed structure under higher sliding velocity and normal load are much larger than those in reference [27] due to different structural design and piezoelectric mechanism.

In addition, the influences of $$\mu _{S} $$, $$\mu _{D} $$, *C* and $$F_{N} $$ on the system power generation is shown in Fig. 3b. It can be seen from Fig. 3b that the change of these parameters has little effect on the power generation of the system with other parameters remaining unchanged. The increments of $$F_{N} $$ and $$\mu _{S} $$ lead to higher power generation, while with higher values of *C* and $$\mu _{D} $$, the system power generation becomes smaller. It is noted that the power generation of the system comes from the work of state conversion between periodic stick and sliding friction. Larger $$F_{N} $$ and $$\mu _{S} $$ values introduce the greater static friction force during the stick state, but greater values of *C* and $$\mu _{D} $$ lead to less difference between the static and sliding friction forces as well as smaller dynamic work done by the friction.

### Bi-linear Analysis

**Fig. 4:**
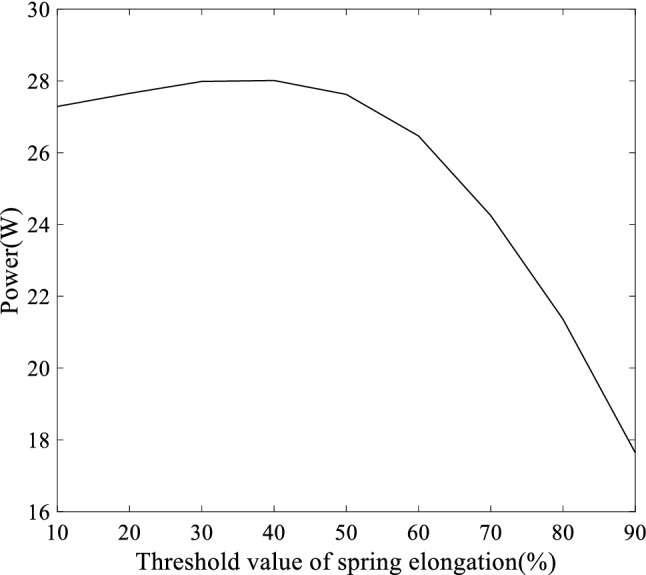
Influence of bilinear spring elongation threshold on power generation ($F_{N} =$10000 N, $\mu _{S} =$0.5, $\mu _{D} =$0.2, $\xi =$0.001, $C=$6, $v=$9 m/s, $k=0.8\times 10^{6}$-$1.4\times 10^{6}$ N/m, and $m=$3 kg)

On the basis of previous research, the elastic bi-linearity is introduced to study its effect on power generation by changing $k'$, when the elongation of the tension spring reaches a certain value (the threshold value), and the equivalent stiffness of the whole system, *k*, changes. The average power generation and maximum mass block displacement of the linear single-degree-of-freedom shear system are 11.96 w and 0.02 m, respectively, with the parameters $F_{N} =$10000 N, $\mu _{S} =$0.5, $\mu _{D} =$0.2, $\xi =$0.001, $C=$6, $v=$9 m/s, $k=0.8\times 10^{6}$ N/m, and $m=$3 kg. For parameter study, with other parameters remaining unchanged, the threshold changes from 10% to 90% of the linear system displacement (0.02 m) with the value of *k* jumping from $0.8\times 10^{6}$ N/m to $1.4\times 10^{6}$ N/m. The variation trend of power generation of the system with the change of threshold value is shown in Fig. 4. It can be seen from Fig. 4 that the power generation of the bi-linear system is higher than that of the linear piezoelectric shear system. The power generation of the system increases first and then decreases with the increase of the threshold value of mass block displacement when bi-linearity occurs. For bi-linear systems with all other parameters as constants, the highest power generation can be obtained by choosing the appropriate threshold of mass block displacement.

## Conclusion

This paper proposes and describes an electrical power generation device using high-frequency dynamic piezoelectric shear deformation under friction. Through parameter studies, the power generation increases with the increases of the equivalent mass of the system, normal pressure, and moving velocity of the top plate. More significant difference between the static friction coefficient and its lower limit will benefit the power generation. Furthermore, possible elastic bi-linearity of the piezoelectric structure can help to generate higher power generation, and an optimized threshold of elastic deformation, where the system stiffness changes, will lead to the highest power generation. For the system with a plate moving at a constant top velocity of 15 m/s on the top of the piezoelectric patch, the proposed device can generate electrical power up to 50 Watt. In future work, we will follow up with experimental realization when materials and equipment are available.

## Figures and Tables

**Fig. 5:**
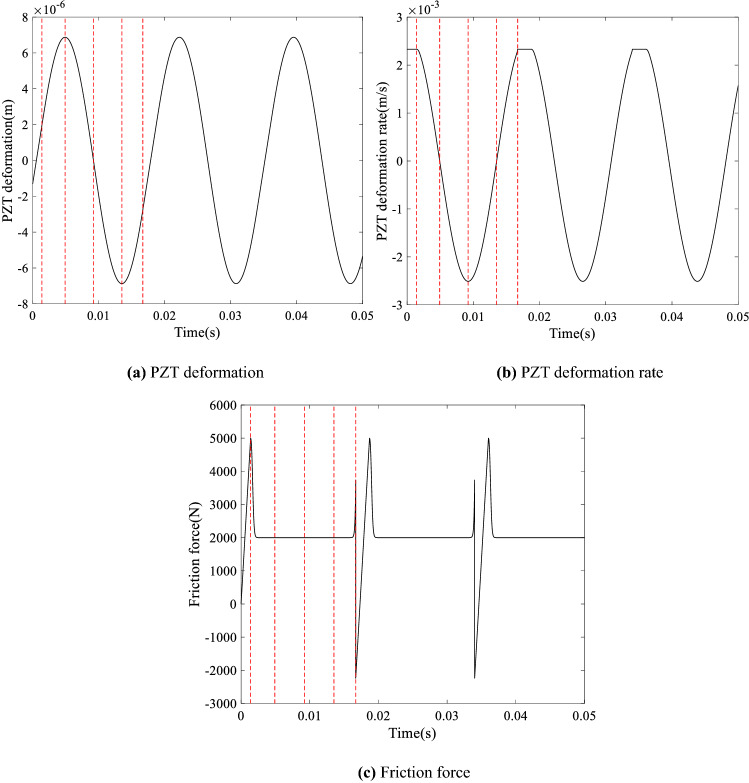
Friction transformation process ($v=$9 m/s, $k=0.4\times 10^{6}$ N/m, $m=$3 kg, $F_{N} =$10000 N, $\mu _{S} =$0.5, $\mu _{D} =$0.2, $\xi =$0.001, $C=$6)

## Appendix A

Before studying the power generation of the proposed PZT shear system, firstly, the dynamic characteristics of the PZT under friction are briefly explored in this sub-section. The mechanical and electrical material properties and dimensions of piezoelectric patch (APC 850) used in the numerical calculation are set to be: $K^\mathrm{T}_{33} =1900$, $d_{15} =5.9\times 10^{-10}$ C/N, $\gamma _{1} =6.3\times 10^{10}$ N/m$^{2}$, $\nu _{13} =0.36$, $l=0.2$m, $w=0.02$ m and $h=0.06$m. Considering the proposed dimension of the PZT patch and an equivalent spring stiffness of $k=0.4\times 10^{6}$ N/m, with the elastic clamp connected to the PZT in series, the PZT shear deformation away from its vibration equilibrium position under friction, deformation rate, friction force and transformation process between sliding and stick friction states within 0-0.05 s are obtained and shown in Fig. 5. All other constants in the simulation are given in the figure caption with a relatively small friction force delay coefficient $C=$6 to consider a gradually friction force variation when the friction state changes. This specific equivalent stiffness is selected to make the stick time longer, which is convenient to explain the transformation process between stick and sliding friction status.

From the results in Fig. 5, it is seen that during $t=$0-0.001389 s, the PZT and the plate are in the stick state, and the PZT and the plate move at a constant velocity simultaneously. The stick friction and the PZT deformation both increases linearly with time. From $t=$0.001389 s, the contact state between PZT and plate changes from stick to sliding. In the period of 0.001390-0.004913 s, the PZT deformation increases continuously and reaches a maximum at $t=$0.004913 s. The sliding friction force between the PZT and the plate decreases with the increase of relative velocity between the mass block and top plate. When the time is 0.004913 s, the PZT deformation rate decreases to 0 m/s. Within $t=$0.004914-0.009212 s, the PZT begins to move in negative direction and its deformation rate increases, and the sliding friction applied on PZT decreases continuously. During $t=$0.009213-0.013516 s, the PZT deformation rate begins to decrease in the negative direction, and the friction force increases. At $t=$0.013517 s, the PZT deformation rate decreases to 0 m/s and starts to accelerate to the positive direction. When the time is in the period of 0.013517-0.016692 s, the PZT deformation rate keeps increasing, and the sliding friction continues to increase. At $t=$0.016693 s, the velocity of the mass block and plate is equal, and the spring force is less than the maximum static friction at this time, the contact state between the PZT and the plate turns to stick.

## References

[1] Lü C, Zhang Y, Zhang H (2019). Influences of environmental motion modes on the efficiency of ultrathin flexible piezoelectric energy harvesters. Acta Mech Solida Sin.

[2] Hu Y, Hu T, Jiang Q (2007). Coupled analysis for the harvesting structure and the modulating circuit in a piezoelectric bimorph energy harvester. Acta Mech Solida Sin.

[3] Zhang YW, Wang SL, Ni ZY (2019). Integration of a nonlinear vibration absorber and levitation magnetoelectric energy harvester for whole-spacecraft systems. Acta Mech Solida Sin.

[4] Aladwani A, Aldraihem O, Baz A. Single degree of freedom shear-mode piezoelectric energy harvester. J Vib Acoust. 2013; 135(5).

[5] Qin L, Jia J, Choi M (2019). Improvement of electromechanical coupling coefficient in shear-mode of piezoelectric ceramics. Ceram Int.

[6] Kulkarni V, Ben-Mrad R, Prasad SE (2013). A shear-mode energy harvesting device based on torsional stresses. IEEE ASME Trans Mechatron.

[7] Majidi C, Haataja M, Srolovitz DJ (2010). Analysis and design principles for shear-mode piezoelectric energy harvesting with ZnO nanoribbons. Smart Mater Struct.

[8] Wang DA, Liu NZ (2011). A shear mode piezoelectric energy harvester based on a pressurized water flow. Sens Actuator Phys.

[9] Narolia T, Gupta VK, Parinov IA (2020). Design and analysis of a shear mode piezoelectric energy harvester for rotational motion system. J Adv Dielectr.

[10] Malakooti MH, Sodano HA. Shear mode energy harvesting of piezoelectric sandwich beam. Proc SPIE. 2013;8688(3):86881R-86881R-13.

[11] Zheng X, Zhang Z, Zhu Y (2014). Analysis of Energy Harvesting Performance for Mode Piezoelectric Bimorph in Series Connection Based on Timoshenko Beam Model. IEEE ASME Trans Mechatron.

[12] Malakooti MH, Sodano HA (2015). Piezoelectric energy harvesting through shear mode operation. Smart Mater Struct..

[13] Zeng Z, Ren B, Gai L, et al. Shear-mode-based cantilever driving low-frequency piezoelectric energy harvester using 0.67 Pb (Mg 1/3 Nb 2/3) O 3-0.33 PbTiO 3. IEEE Trans Ultrason Ferroelectr Freq Control. 2016;63(8):1192–1197. 10.1109/TUFFC.2016.2572308 27244735

[14] Zhou L, Sun J, Zheng XJ (2012). A model for the energy harvesting performance of shear mode piezoelectric cantilever. Sens Actuator Phys.

[15] Huang GY, Yan JF (2017). A mechanical model for the adhesive contact with local sliding induced by a tangential force. Acta Mech Solida Sin.

[16] La S, Wang J, Zhang X (2018). Behavior of a Micro-sized Superconducting Fiber in a Low-Temperature Medium: Experimental and Computational Analysis [J]. Acta Mech Solida Sin.

[17] Nakano K, Maegawa S (2009). Safety-design criteria of sliding systems for preventing friction-induced vibration. J Sound Vib.

[18] Graf M, Ostermeyer GP (2015). Friction-induced vibration and dynamic friction laws: instability at positive friction-velocity-characteristic. Tribol Int.

[19] Kruse S, Tiedemann M, Zeumer B (2015). The influence of joints on friction induced vibration in brake squeal. J Sound Vib.

[20] Wang DW, Mo JL, Wang XF (2018). Experimental and numerical investigations of the piezoelectric energy harvesting via friction-induced vibration. Energy Convers Manag.

[21] Stender M, Tiedemann M, Hoffmann N (2019). Energy harvesting below the onset of flutter. J Sound Vib.

[22] Li Q (2016). Limiting profile of axisymmetric indenter due to the initially displaced dual-motion fretting wear. FU Mech Eng.

[23] Li Z, Ouyang H, Guan Z. Nonlinear friction-induced vibration of a slider–belt system. J Vib Acoust. 2016;138(4).

[24] Xing P, Li G, Gao H (2020). Experimental investigation on identifying friction state in lubricated tribosystem based on friction-induced vibration signals. Mech Syst Signal Process.

[25] Green PL, Worden K, Sims ND (2013). On the identification and modelling of friction in a randomly excited energy harvester. J Sound Vib.

[26] Wang DW, Liu MX, Wu X, et al. Investigation of Piezoelectric Energy Harvesting via Nonlinear Friction-Induced Vibration. Shock Vib. 2020.

[27] Tadokoro C, Matsumoto A, Nagamine T (2017). Piezoelectric power generation using friction-induced vibration. Smart Mater Struct.

[28] Li Y, Yin D, Cheng X (2020). Vibration energy harvesting with piezoelectric ceramics working in d33 mode by using a spring-mass-spring oscillator. J Appl Phys..

[29] Chen W, Xiang ZY, Mo JL (2020). Energy harvesting and vibration reduction by sandwiching piezoelectric elements into elastic damping components with parallel-grooved structures. Compos Struct.

[30] Wang DW, Liu MX, Qian WJ, et al. Parametrical investigation of piezoelectric energy harvesting via friction-induced vibration. Shock Vib. 2020.

[31] Mostaghel N, Byrd RA (2000). Analytical description of multidegree bilinear hysteretic system. J Eng Mech.

[32] Shi B, Yang J, Rudd C (2019). On vibration transmission in oscillating systems incorporating bilinear stiffness and damping elements. Int J Mech Sci.

[33] Tien MH, D’Souza K. Analyzing bilinear systems using a new hybrid symbolic-numeric computational method. J Vib Acoust. 2019;141(3).

